# Dense‐core vesicle biogenesis and exocytosis in neurons lacking chromogranins A and B

**DOI:** 10.1111/jnc.14263

**Published:** 2017-12-27

**Authors:** Natalia Dominguez, Jan R. T. van Weering, Ricardo Borges, Ruud F. G. Toonen, Matthijs Verhage

**Affiliations:** ^1^ Department of Clinical Genetics Center for Neurogenomics and Cognitive Research (CNCR) VU University Amsterdam and VU University Medical Center (VUmc) Amsterdam The Netherlands; ^2^ Unidad de Farmacología Facultad de Medicina Universidad de la Laguna Tenerife Spain; ^3^ Functional Genomics Center for Neurogenomics and Cognitive Research (CNCR) VU University Amsterdam and VU University Medical Center (VUmc) Amsterdam The Netherlands

**Keywords:** exocytosis, live‐cell imaging, neuromodulation, neuropeptides, neurotransmission, secretion

## Abstract

Chromogranin A and B (Cgs) are considered to be master regulators of cargo sorting for the regulated secretory pathway (RSP) and dense‐core vesicle (DCV) biogenesis. To test this, we analyzed the release of neuropeptide Y (NPY)‐pHluorin, a live RSP reporter, and the distribution, number, and appearance of DCVs, in mouse hippocampal neurons lacking expression of CHGA and CHGB genes. qRT‐PCR analysis showed that expression of other granin family members was not significantly altered in CgA/B^−/−^ neurons. As synaptic maturation of developing neurons depends on secretion of trophic factors in the RSP, we first analyzed neuronal development in standardized neuronal cultures. Surprisingly, dendritic and axonal length, arborization, synapse density, and synaptic vesicle accumulation in synapses were all normal in CgA/B^−/−^ neurons. Moreover, the number of DCVs outside the soma, stained with endogenous marker Secretogranin II, the number of NPY‐pHluorin puncta, and the total amount of reporter in secretory compartments, as indicated by pH‐sensitive NPY‐pHluorin fluorescence, were all normal in CgA/B^−/−^ neurons. Electron microscopy revealed that synapses contained a normal number of DCVs, with a normal diameter, in CgA/B^−/−^ neurons. In contrast, CgA/B^−/−^ chromaffin cells contained fewer and smaller secretory vesicles with a smaller core size, as previously reported. Finally, live‐cell imaging at single vesicle resolution revealed a normal number of fusion events upon bursts of action potentials in CgA/B^−/−^ neurons. These events had normal kinetics and onset relative to the start of stimulation. Taken together, these data indicate that the two chromogranins are dispensable for cargo sorting in the RSP and DCV biogenesis in mouse hippocampal neurons.

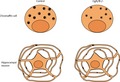

**Abbreviations used**
a.u: arbitrary units
CgA: chromogranin A
CgB: chromogranin B
Cgs: chromogranin A and B
CNS: central nervous system
DCV: dense‐core vesicle
DIV: day *in vitro*
EM: electron microscopy
GFP: green fluorescence protein
HBSS: Hanks' balanced salt solution
KO: knock‐out
MAP2: microtubule‐associated protein 2
NPY: neuropeptide Y
PBS: phosphate‐buffered saline
RSP: regulated secretory pathway
Sg: secretogranin
Syp1: synaptophysin1

Neurons communicate via fast neurotransmission (milliseconds) mediated by synaptic vesicle exocytosis and via the release of neuropeptides, neurotrophic factors, and guidance cues with slower and longer lasting effects (seconds to minutes). These neuromodulators are involved in brain development and synaptic plasticity, and influence behavior (see McAllister *et al*. 1995; Poo 2001; Dickson 2002; van den Pol 2012). Impairment of neuromodulator function has been linked to disorders such as autism, social anxiety disorder, schizophrenia, major depression, and obesity (Meyer‐Lindenberg *et al*. 2011; Guilloux *et al*. 2012; Morello *et al*. 2016). Neuropeptides are packaged in dense‐core vesicles (DCVs), which bud from the Trans‐Golgi Network as immature vesicles. During DCV maturation, missorted proteins are removed, the intravesicular milieu is acidified and the cargo is condensed (Kim *et al*. 2006; Dikeakos and Reudelhuber 2007). DCVs are transported through axons and dendrites (Lo *et al*. 2011; Lipka *et al*. 2016) and released in a calcium‐dependent manner upon high‐frequency stimulation from synaptic and extrasynaptic sites (de Wit *et al*. 2009; van de Bospoort *et al*. 2012; Farina *et al*. 2015).

Chromogranin A (CgA) and Chromogranin B (CgB) are members of the Chromogranin/Secretogranin family, also known as granins. In the text, Chromogranins (Cgs) refers only to CgA and CgB but not the rest of the granins. CHGA and CHGB are two related genes, abundantly expressed in neurosecretory tissue that are proposed to act as master regulators of DCV formation by promoting the aggregation of cargo destined for the regulated secretory pathway (RSP) and initiating the sorting of such cargo relative to constitutive secretion (Huttner and Natori 1995; Natori and Huttner 1996; Krömer *et al*. 1998; Montero‐Hadjadje *et al*. 2009; Sun *et al*. 2013). Cgs are the most abundant constituents of DCVs intravesicular matrix (Taupenot *et al*. 2003; Montero‐Hadjadje *et al*. 2008) that bind Ca^2+^ and aggregate at acidic pH (Yoo and Albanesi 1991; Videen *et al*. 1992). This aggregation ability is considered to be a driving force for DCV biogenesis. Heterologous Cgs expression induced granule‐like structures in cell lines without a RSP (Kim *et al*. 2001; Huh *et al*. 2003; Stettler *et al*. 2009; Dominguez *et al*. 2014), whereas Cgs knock down in cell lines with a RSP decreased the number of DCVs (Kim *et al*. 2001; Huh *et al*. 2003; Courel *et al*. 2010). A reduction in the number and volume of chromaffin granules was found in a mouse line lacking CgA (Mahapatra *et al*. 2005; Pasqua *et al*. 2016) and antisense‐CgA transgenic mice showed enlarged and reduced number of chromaffin DCVs (Kim *et al*. 2005). Together, these data led to the conclusion that Cgs are ‘on/off’ switches controlling DCV biogenesis (Kim *et al*. 2001; Huh *et al*. 2003). However, no changes in DCV biogenesis and morphology were found in a different CgA^−/−^ line (Hendy *et al*. 2006), which was explained by the up‐regulation of CgB in these mice (Hendy *et al*. 2006). CgB^−/−^ mice showed no changes in the number and morphology of insulin granules in β‐cells, whereas the number of DCVs docked in pituitary adenocorticotropic cells was reduced by 30% (Obermüller *et al*. 2010). Genetic inactivation of both CHGA and CHGB genes in mice showed a 34% reduction in the number of release events in chromaffin cells, an altered morphology of the chromaffin granules, a reduced catecholamine content, and altered kinetics of catecholamine release (Montesinos *et al*. 2008; Díaz‐Vera *et al*. 2010, 2012). Hence, Cgs' role in cargo sorting, DCV biogenesis, and fusion has been established in different endocrine cells, showing some consistent and some contradictory results. However, their role in cargo sorting, DCV biogenesis, and fusion has not been assessed in CNS neurons. The other members of the Chromogranin/Secretogranin family are: Secretogranin II (SgII), Secretogranin III (SgIII), Secretogranin V (SgV) or 7B2, Secretogranin VI (SgVI) or NESP, VGF, proSAAS (Bartolomucci *et al*. 2011). Several of them have been also involved in DCV biogenesis as SgII, SgIII, and VGF (Courel *et al*. 2010; Sun *et al*. 2013; Fargali *et al*. 2014).

In this study, we evaluated the role of Cgs in cargo sorting, DCV biogenesis, and fusion in hippocampal neurons. We used CgA/B^−/−^ hippocampal neurons expressing a pH‐sensitive fluorescent probe for the RSP, to visualize cargo loading, vesicular acidification, and exocytotic events. Surprisingly, we found that the lack of both Cgs did not affect these parameters in hippocampal neurons.

## Methods

### Ethical statement

Animals were housed, handled, and bred according to institutional, Dutch and EU governmental guidelines and to approved VU University Animal Ethics and Welfare Committee protocols (Ethical approval reference number: FGA 11‐06). Mice were housed as follows: in type 2 enlarged cage, maximum of three adult mice per cage, light/dark cycle of 12 h each, 20–22°C, water and food *ad libitum*, with environmental enrichment. Postnatal day 1 mice with an average weight 1–1.5 g were killed immediately after separation from the mother by decapitation ensuring minimal suffering of the animals. The two hippocampi of one mice of each genotype were used for each independent primary culture. Cultured neurons were analyzed both by live‐cell imaging experiments and immunofluorescence staining allowing the reduction in the number of animals used. Experimental procedures were performed without blinding. Imaging analysis was performed in a blind fashion, aleatory numbers were assigned to the images and movies, the experimental groups were revealed after the analysis. The study was not pre‐registered.

### Animals

CgA/B^−/−^ (RRID: MGI:5919969) mice were sourced from Dr. Ricardo Borges' laboratory. Their generation and backcross to C57Bl/6J background were described previously (Díaz‐Vera *et al*. 2012; Pereda *et al*. 2015). Mice were backcrossed two extra generations into a C57Bl/6J background at the VU University Amsterdam. C57Bl/6J (RRID: IMSR_JAX:000664) mice were obtained from Charles River Laboratories Inc., Sulzfeld, Germany. Hippocampal neurons for primary culture were obtained from postnatal day 1 littermates of both sexes after decapitation. Mice were genotyped and double knock‐out (KO) mice were compared with double heterozygous mice, which were used as control. No randomization was performed, mice were assigned to group double KO or double heterozygous by their genotype.

### Primary cultures

Hippocampal neurons were cultured on micro‐islands of glia as described in (Farina *et al*. 2015). Briefly, 18‐mm coverslips were etched and coated with agarose (Type II‐A; Sigma Aldrich, Zwijndrecht, The Netherlands) and islands were stamped with a solution of 0.1 mg/mL poly‐d‐lysine (Sigma Aldrich) and 0.73 mg/mL rat tail collagen I (Corning, Amsterdam, The Netherlands) in 10 mM acetic acid (VWR). Rat glia was plated in a density of 6000 cells/well in Dulbecco's modified Eagle's medium (Gibco, Rockville, MD, USA). Hippocampi from P1 mice were dissected in Hanks' balanced salt solution (Sigma Aldrich) and digested with 0.25% trypsin (Gibco) for 20 min at 37°C. Hippocampi were washed with Hanks' balanced salt solution and triturated with fire‐polished pipettes. Neurons were counted and plated at a density of 1500 neurons/well in 12‐well plates with Neurobasal medium (Gibco) supplemented with 2% B‐27 (Gibco), 1.8% HEPES (Gibco), 1% glutamax (Gibco), and 1% Penicillin‐Streptomycin (Gibco).

### Lentiviral transduction

Neuropeptide Y (NPY)‐pHluorin plasmid was previously generated (de Wit *et al*. 2009) and subcloned into a pLentiviral vector with a synapsin promoter to specifically express the probe in neurons. Lentiviral particles were produced as described before (Naldini *et al*. 1996). Neurons were infected at days *in vitro* (DIV)10 and imaged at DIV18–21.

### Western blot

Samples from whole brain and whole adrenal glands from P1 mice were separated by sodium dodecyl sulfate–polyacrylamide gel electrophoresis in 8% acrylamide gels and electroblotted onto 0.22 μm polyvinylidene difluoride membranes (Bio‐Rad Laboratories, Veenendaal, The Netherlands). Membranes were probed with the following primary antibodies: rabbit polyclonal anti‐CgA (Cat# 259 003, RRID: AB_2619972; Synaptic Systems, Goettingen, Germany), rabbit polyclonal anti‐CgB (Cat# 259 103, RRID: AB_2619973; Synaptic Systems), and mouse monoclonal anti‐actin (Cat# MAB1501, RRID: AB_2223041; Millipore Corporation, Bedford, MA, USA). Alkaline phosphatase‐conjugated antibodies (Jackson Immuno‐Research, West Grove, PA, USA) were used to visualize the protein bands using the AttoPhos^®^ AP Fluoresent Substrate System (Promega, Madison, WI, USA). Membranes were scanned with a FLA‐5000 Fujifilm device and analyzed with the software ImageJ (NIH, Bethesda, Maryland, USA).

### Quantitative RT‐PCR

Total RNA was extracted from whole brains using UltraClean^®^ Tissue & Cells RNA Isolation kit (15000‐50; MO BIO, Carlsbad, CA, USA). Synthesis of cDNA was performed using oligo d(T) and random hexamers with the kit iScriptTM select cDNA Synthesis Kit (1708896; BIO‐RAD, Madrid, Spain). Quantitative RT‐PCR was performed with SensiFastTM SYBR^®^ Lo‐Rox kit (BIO‐94005; BIOLINE, London, UK) using de Light Cycler 480 System (Roche Applied Science, Woerden, The Netherlands) with the following primers: SCG2‐F: GCTGTCCGGTGCTGAAA, SCG2‐R: TTAGCTCCAGCCATGTCTTAAA, SCG3‐F: ACCCTGGATAAACCCACAAG, SCG3‐R: CACGGTTAGTGAAGCCATCT, SCG5‐F: TGGGCAAGTGGAACAAGAA, SCG5‐R: GTCCAACCTCTTTCCTTGTAGAT, GNAS‐F: CGCTGCAAGACCAGGAG, GNAS‐R: CAGACTCTCCAGCACCTTTATC, VGF‐F: AGGCTCGAATGTCCGAAAG, VGF‐R: TGACACCGGCTGTCTCT, PCSK1N‐F: TTTGGTGCTGCTGCTCTT, PCSK1N‐R: GAGTGCTCGTCTCAACCAAG, eEF2a‐F: CAATGGCAAAATCTCACTGC, eEF2a‐R: AACCTCATCTCTATTAAAAACACCAAA. eEF2a was used as reference gene for normalizing the data across samples. The 2^−ΔΔCt^ method was used for calculating the fold change in expression of the different genes.

### Live‐cell imaging

Coverslips with neurons expressing NPY‐pHluorin were placed in a chamber and perfused with Tyrode's solution (119 mM NaCl, 2.3 mM KCl, 2 mM CaCl_2_, 2 mM MgCl_2_, 25 mM HEPES, 30 mM Glucose, pH 7.4) at 18 ‐ 23°C. Live‐cell imaging experiments were carried out on an inverted microscope (IX81; Olympus, Leiderdorp, The Netherlands) equipped with an illumination unit (MT20; Olympus), appropriate filter cube sets (Semrock, Inc, Rochester, NY, USA), a 40× oil objective (NA 1.3), and an EM‐CCD camera (C9100‐02; Hammamatsu Photonics, Almere, The Netherlands). Xcellence RT software controlled the microscope and the image acquisition. Each neuron was imaged for 90 s with an acquisition frequency of 2 Hz. DCV fusion was elicited by electrical field stimulation. Platinum electrodes were placed surrounding an island containing a single neuron. The stimulation protocol was controlled by a Master‐8 system (AMPI, Jerusalem, Israel) and electric pulses of 1 ms and 30 mA were delivered by a stimulus generator (A385C; WPI, Berlin, Germany). The stimulation protocol used was 16 trains of 50 action potentials at 50 Hz with 0.5 s interval (16 × 50AP@50 Hz). The imaging protocol consisted of 30 s baseline, 23.5 s stimulation (16 × 50AP@50 Hz), 26.5 s recovery period followed by 5 s superfusion of 50 mM NH_4_Cl Tyrode's solution (NaCl was replaced in a equimolar way by NH_4_Cl) and 5 s recovery. NH_4_Cl was applied by a barrel system to unquench NPY‐pHluorin by neutralizing the intravesicular pH, allowing the quantification of the total pool of DCVs (see Image analysis for more details). Quality of the neuronal culture was assessed by Ca^2+^ dynamics measurements (using Fluo5F‐AM; Molecular Probes, Eugene, OR, USA) applying the same stimulation protocol used to trigger DCV fusion. Cells were included for DCV fusion experiments when the Ca^2+^ signal sharply increased during the stimulation and returned to baseline in the 10 s after (Figure S1).

### Immunocytochemistry

DIV16–21 hippocampal neurons from both genotypes were fixed in 4% paraformaldehyde solution (Electron Microscopy Sciences, Hatfield, PA, USA) in phosphate‐buffered saline (PBS: 137 mM NaCl, 2.7 mM KCl, 10 mM Na_2_HPO_4_, 1.8 mM KH_2_PO_4_) pH 7.4, at 18 ‐ 23°C for 20 min and washed with PBS afterward. Cells were permeabilized in PBS containing 0.5% Triton X‐100 (Fisher Chemical, Landsmeer, The Netherlands) for 5 min and then incubated for 30 min with PBS containing 2% normal goat serum and 0.1% Triton X‐100. Primary antibodies were incubated for 2 h and secondary antibodies were incubated for 1 h, both at 18 ‐ 23°C. Primary antibodies used were: guinea pig polyclonal synaptophysin1 (Syp1) (Cat# 101 004, RRID:AB_1210382; Synaptic Systems), chicken polyclonal microtubule‐associated protein 2 (MAP2) (Cat# ab5392 RRID:AB_2138153; Abcam, Cambridge, UK), mouse monoclonal SMI312 (Cat# 837904, RRID: AB_2566782; Biolegend, London, UK), rabbit polyclonal Secretogranin II (SgII) (Cat# K55101R RRID:AB_152813; Meridian Life Science, Memphis, TN, USA) and rabbit polyclonal green fluorescence protein (Cat# GTX20290 RRID:AB_371415; GeneTex, Irvine, CA, USA). Alexa Fluor conjugated secondary antibodies were from Invitrogen, Carlsbad, CA, USA. Coverslips were mounted in Mowiol (Sigma Aldrich) and Z‐stacks of single neurons were acquired on a confocal laser‐scanning microscope (LSM 510; Carl Zeiss, Breda, The Netherlands) with a 40× objective (NA = 1.3).

### Image analysis

**Figure 1:**
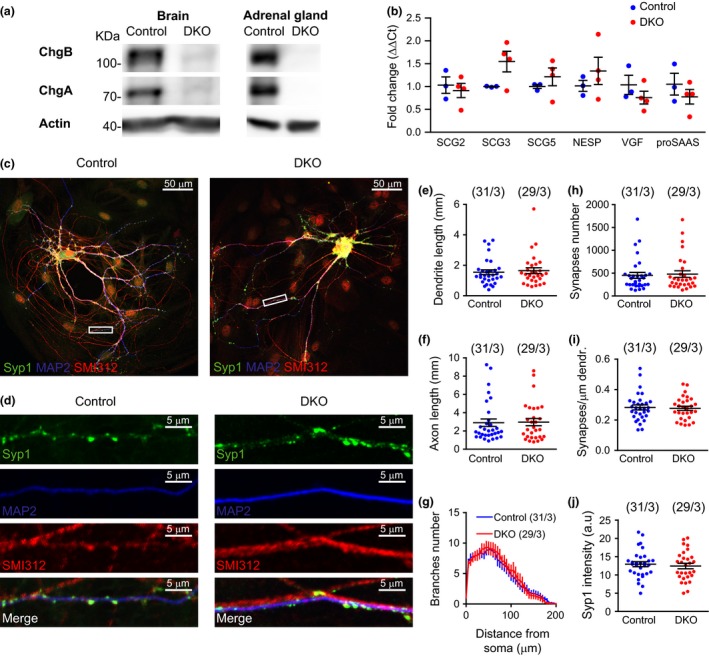
Neuronal morphology of hippocampal neurons is normal in the absence of Cgs. (a) Western blot showing the absence of Cgs in brain and adrenal gland lysates from WT and CgA/B^−/−^ P1 mice. (b) qRT‐PCR analysis showing the fold change expression of different members of the Secretogranin family. (c) Example images of control and CgA/B^−/−^ hippocampal neurons (DIV19) stained for the synapse marker Syp1 (green), the dendrite marker microtubule‐associated protein 2 (MAP2) (blue) and the axonal marker SMI312 (red). SMI‐positive (red) nuclei are the astrocytes nuclei, known to contain SMI immunoreactivity (Weigum *et al*. 2003). (d) Zoomed areas in the boxes shown in (b). (e) Quantification of the dendritic length (MAP2). (f) Quantification of the axonal length (SMI312). (g) Sholl analysis showing the mean number of dendritic branches against the distance from soma. (h) Quantification of the number of synapses (synaptophysin1 puncta on the dendrites) per neuron. (i) Quantification of the number of synapses per μm dendrite per neuron. (j) Quantification of synaptophysin1 intensity per synapse per neuron. (Data shown as mean ± SEM. (b) Control *n* = 3 mice and CgA/B^−/−^ *n* = 4 mice MW‐U test. (e–j). Control *n* = 31 neurons and CgA/B^−/−^ *n* = 29 neurons from three independent cultures. (e–i) MW‐U test. (j) Student's *t*‐test.

**Figure 2:**
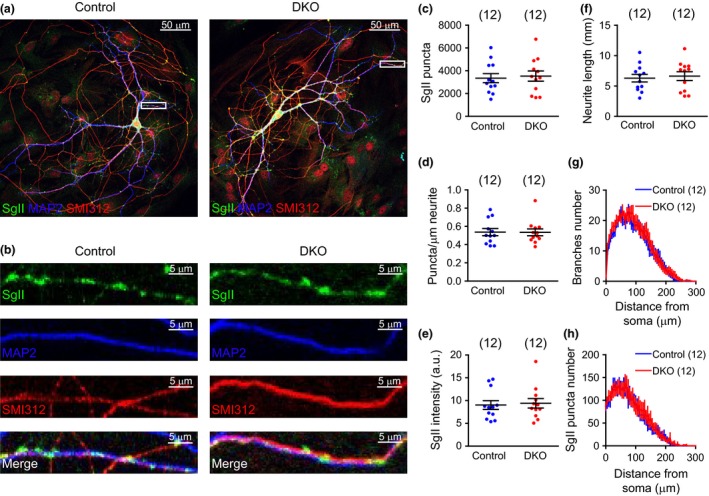
SgII puncta number and intensity are normal in the absence of Cgs. (a) Example images of control and CgA/B^−/−^ hippocampal neurons (DIV16) stained for the dense‐core vesicle marker SgII (green), the dendritic marker microtubule‐associated protein 2 (MAP2) (blue) and the axonal marker SMI312 (red). SMI‐positive (red) nuclei are the astrocytes nuclei, known to contain SMI immunoreactivity (Weigum *et al*. 2003). (b) Zoomed areas in the boxes shown in (a). (c) Quantification of the number of SgII puncta per neuron. (d) Quantification of the number of SgII puncta per μm per neurite. (e) Mean intensities of the SgII puncta per neuron. (f) Analysis of the total neurite length (MAP2 and SMI312 staining). (g) Sholl analysis showing the mean number of neurite branches against the distance from soma. (h) Analysis of the mean number of SgII puncta against the distance from soma. (Data shown as mean ± SEM. Control *n* = 12 neurons and CgA/B^−/−^ *n* = 12 neurons. (c and f) Student's *t*‐test, (d and e) MW‐U test.

Quantification of the length and branching of axons (SMI312 staining) and dendrites (MAP2 staining), number of synapses (Syp1), synapses area (Syp1), number of SgII or NPY‐pHluorin puncta, fluorescence intensity of the puncta, in Figs 1(c and d), 2(a and b), and 4(e and f) were performed with the automated image analysis software SynD described in Schmitz *et al*. 2011 running in MATLAB (MathWorks, Inc, Natick, MA, USA). Maximum projections of confocal Z‐stacks were generated using ImageJ and analyzed with the software SynD (Schmitz *et al*. 2011). MAP2 and/or SMI312 were used by the software to create automatically a mask of the neuronal morphology. With this mask, the software measured dendritic and axonal length and performed a Sholl analysis to calculate the branching of the neurons. Subsequently, the number of synapses stained with Syp1 (Fig. 1b and c) or DCVs stained with SgII (Fig. 2a and b) or labeled with over‐expressed NPY‐pHluorin (Fig. 4e and f) were detected, counted and the intensity of the signal was measured automatically by the software.

Colocalization of SgII and NPY‐pHluorin in Fig. 4 was analyzed by the calculation of Pearson's and Mander's coefficients using the ImageJ plugin JaCoP described in (Bolte and Cordelières 2006).

Live‐cell imaging recordings were analyzed with ImageJ software. Fusion events were located by eye and 3 × 3 pixel (0.6 × 0.6 μm) regions of interest were placed centered on the position of the event. NPY‐pHluorin signal is not quenched in the Golgi because the intraluminal pH is not acidic; yet, consequently, the measurement of fusion events in the soma is not reliable and somatic events were therefore excluded. Fluorescence intensity of individual events was measured over time of acquisition using the ImageJ plugin Time Series Analyzer and plotted as fluorescence increase (ΔF) compared to the initial fluorescence signal (F_0_). Fusion events were included in the analysis when a sharp increase in fluorescence above two times SD of the background was observed.

The duration of the fusion events was the time between the first frame when the signal was above two times the background SD, and the frame when the signal was again below two times the background SD. If the signal did not go back to background, the end point of the event was the start of the NH_4_Cl puff. Depending on the duration of the events, they were classified in four categories: short transient (≤ 1 s), long transient (> 1 s to ≤ 5 s), short persistent (> 5 s to ≤ 10 s), and long persistent (> 10 s).

An estimation of the total pool of DCVs of each neuron was calculated using the NH_4_Cl puff during the live‐cell imaging experiments. Maximum projections of the NH_4_Cl images were obtained and the first frame of the movie was subtracted (using Image J software) to remove signal coming from non‐acidic compartments. A mask of the neurites was created, and the number and intensity of NPY‐pHluorin puncta were automatically quantified as described before using SynD software. DCVs can occasionally travel in clusters of two to five vesicles (van de Bospoort *et al*. 2012) and, therefore, some of the detected puncta may correspond to multiple instead of single DCVs. To avoid underestimation of the vesicular pool, we considered that puncta with values of fluorescence multiplying that of the modal intensity (used as unbiased cutoff) contain a number of DCVs equal to the multiplying factor (Figure S2a).

#### Electron microscopy

Primary hippocampal neuron cultures (DIV16–21) from CgA/B^−/−^ and control mice grown on glass coverslips and adrenal glands were fixed for 90 min at 18 ‐ 23°C with 2.5% glutaraldehyde in 0.1 M cacodylate buffer, pH 7.4. Samples were washed and post‐fixed for 1 h at 18 ‐ 23°C with 1% OsO_4_/1% KRu(CN)_6_. After dehydration through a series of increasing ethanol concentrations, samples were embedded in Epon and polymerized for 48 h at 60°C. For cultured neurons, the coverslip was removed by alternately dipping it in hot water and liquid nitrogen. Of both type of specimens, ultrathin sections (80 nm) were collected on single‐slot formvar‐coated copper grids, and stained in uranyl acetate and lead citrate in Ultra stainer LEICA EM AC20. Synapses and chromaffin cells were randomly selected at low magnification and photographed at 60k× magnification for synapses or 12k× magnification for chromaffin cells using a JEOL1010 transmission electron microscope at 60 kV. The observer was blinded for the genotype. DCVs were recognized by eye as electron dense particles enclosed by a vesicular membrane; they were counted manually and their diameter was quantified as the average of the longest and the shortest diameter of the vesicular membrane structure.

### Statistics

Data are shown as mean ± SEM. Statistical significance between groups was assessed with Student's *t*‐test for data that passed the normality test (D'Agostino Pearson) or Mann–Whitney *U*‐test (MW‐U) for those that did not. The test used for each dataset is specified in the figure legend. Data were analyzed with Prism 5.0 GraphPad Software; GraphPad Software Inc., San Diego, CA, USA. No power analysis was performed for sample size determination. Outliers were not removed from any of the analysis.

## Results

### Morphology and synapse density is normal in CgA/B^−/−^ neurons

Since both single CgA^−/−^ and single CgB^−/−^ mice showed compensatory over‐expression of the other Cg in adrenal glands (Montesinos *et al*. 2008; Díaz‐Vera *et al*. 2010), in this study we used the CgA/B double knock out mice (CgA/B^−/−^), as described before for the analysis of adrenal gland secretory vesicles (Díaz‐Vera *et al*. 2012). The absence of Cgs in the brain and adrenal gland lysates of CgA/B^−/−^ mice was confirmed by western blot (Fig. 1a). Analysis of the expression of the other members of the Chromogranin/Secretogranin family by qRT‐PCR showed no difference in expression between CgA/B^−/−^ and controls, but a trend toward compensatory up‐regulation of SCG3 expression (not significant) (Fig. 1b). Since changes in DCV biogenesis or fusion are expected to have trophic effects on neuronal morphology (axonal/dendritic growth, synapses formation), we first analyzed neuronal morphology and synapse density in DIV18–20 hippocampal neurons from CgA/B^−/−^ and control mice stained for MAP2 (dendritic marker), SMI312 (axonal marker), and Syp1 (synaptic marker; Fig. 1c and d). Images from both genotypes were analyzed creating a mask using SMI312 staining to analyze the axonal length and a different mask for MAP2 to analyze dendritic length and branching using the SynD software (Schmitz *et al*. 2011). The synapses were detected as Syp1‐positive puncta in the MAP2 mask automatically by SynD and different parameters as number of synapses and intensities were quantified. Neither the dendritic length nor the axonal length was different between the two genotypes (Fig. 1e and f). Sholl analysis showed that the dendritic branching of neurons of both genotypes was similar (Fig. 1g). The total number of synapses (quantified as Syp1‐positive puncta on the dendrites) (Fig. 1h) and the number of synapses per μm of dendrite (Fig. 1i) was not altered in CgA/B^−/−^ neurons. Neither the mean intensity of Syp1 per synapse (Fig. 1j) nor the synapse area (data not shown) was altered in the CgA/B^−/−^ neurons meaning that the number of synaptic vesicles and the number of Syp1 molecules per vesicle was the same in both groups. In conclusion, axonal/dendritic growth and synaptogenesis of CgA/B^−/−^ neurons is unaffected by the absence of Cgs.

### DCV biogenesis is normal in the absence of Cgs

To test the effect of Cgs on biogenesis, the number of DCVs per neuron was quantified in CgA/B^−/−^ and control neurons at the light microscopy level on single‐cultured neurons on microdot islands using Secretogranin II (SgII) as endogenous marker, NPY‐pHluorin as heterologous marker (see below), and at the ultrastructural level using electron microscopy (see below). NPY‐pHluorin is a marker for DCVs since it shows an overlap of at least 80% with other reporters if co‐expressed in single neurons, see (de Wit *et al*. 2009; fig. 10b) and Fig. 4(a–d) in this study.

Neurons were stained for SgII, MAP2, and SMI312 (Fig. 2a and b). Subsequently the number of SgII puncta and their intensities per neuron were quantified. Surprisingly, neither the mean number of SgII puncta per neuron (Fig. 2c) nor the mean number of SgII puncta per μm neurite (Fig. 2d) was changed in CgA/B^−/−^ neurons compared to control. The mean SgII signal per punctum was similar between both genotypes (Fig. 2e). No changes in the size (Fig. 2f) or branching (Fig. 2g) of the neurons were found (similar to what was shown in Fig. 1). The distribution of SgII puncta in the neurites was also analyzed as the number of puncta against the distance from the soma (Fig. 2h) and no differences were found between both groups. Taken together, these data show that the number and the loading of DCVs measured using an endogenous maker is not affected by the absence of Cgs.

### DCV size and synaptic distribution are normal in the absence of Cgs

**Figure 3:**
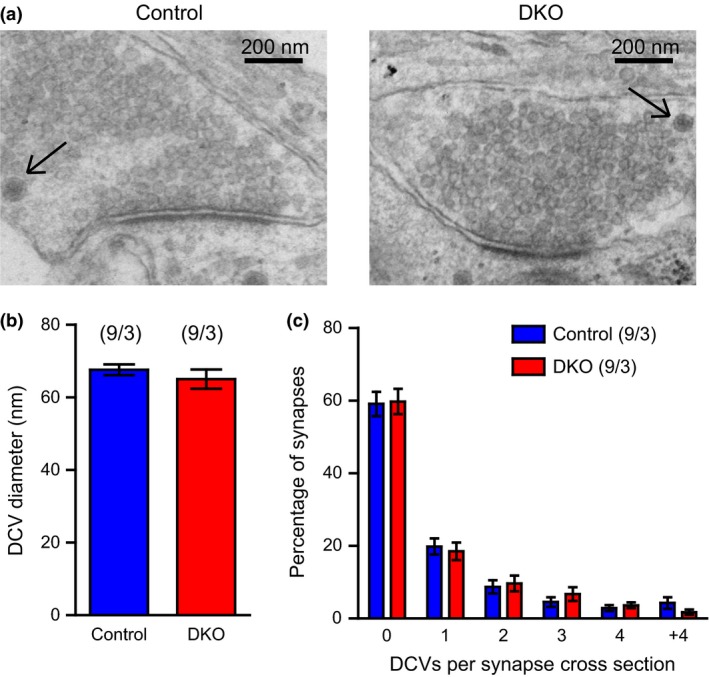
The size and number of synaptic dense‐core vesicles (DCVs) is normal in the absence of Cgs. (a) Example electron micrographs of control and CgA/B^−/−^ hippocampal neurons (DIV19). DCVs are indicated with arrows (b) Quantification of the mean diameter of DCVs. (c) Percentage distribution of the number of DCVs found per synapse. (Data shown as mean ± SEM. For size measurements: Control *n* = 9 neurons (660 DCVs) and CgA/B^−/−^ *n* = 9 neurons (521 DCVs); for DCV distribution in synapses, Control *n* = 9 neurons (377 synapses) and CgA/B^−/−^ *n* = 9 neurons (399 synapses, from three independent cultures). Student's *t*‐test.

Electron microscopy and ultrastructural morphometry were performed to analyze if the size or number of DCVs was modified as consequence of the absence of Cgs. Cultured hippocampal neurons were processed for electron microscopy and imaged for both genotypes (Fig. 3a). CgA/B^−/−^ and control neurons showed DCVs with a similar average diameter (Control: 67.54 ± 1.49 nm and CgA/B^−/−^: 65 ± 2.64 nm) (Fig. 3b). The number of DCVs observed in the synapse cross‐sections of both genotypes was also not changed (Fig. 3c), confirming the conclusions made at the light microscopy level. To confirm published effects of Cgs loss on secretory vesicle morphology in chromaffin cells, we also analyzed secretory vesicles in adrenal gland. In line with some of the previous studies (Mahapatra *et al*. 2005; Pasqua *et al*. 2016), but in contrast to others (Kim *et al*. 2005; Hendy *et al*. 2006; Díaz‐Vera *et al*. 2012), a 23% reduction in secretory vesicle diameter and 40% reduction in the core diameter of the vesicles was observed in adrenal glands of CgA/B^−/−^ mice (Figure S3a–c) and the number of DCVs per chromaffin cell was reduced in CgA/B^−/−^ mice by 20% (Figure S3d). Hence, while previously reported phenotypes in CgA/B^−/−^ chromaffin cells were reproduced, we observed no effects of Cgs loss in hippocampal neurons at the ultrastructural level.

### DCV cargo reporter NPY‐pHluorin indicates normal DCV number, but lower cargo loading in the absence of Cgs

**Figure 4:**
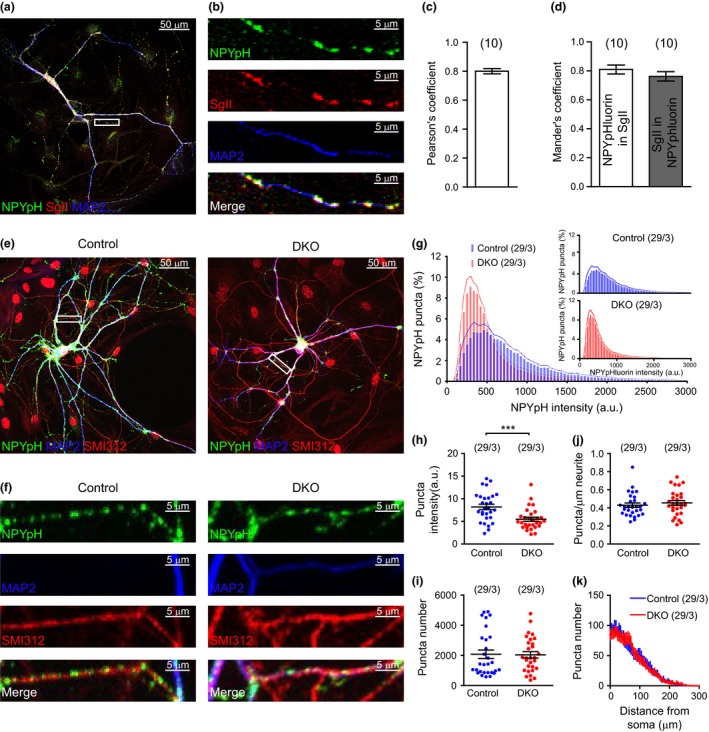
Neuropeptide Y (NPY)‐pHluorin antibody staining intensity is reduced in the absence of Cgs. (a) Example image of WT hippocampal neuron (DIV14) over‐expressing NPY‐pHluorin stained for green fluorescence protein (GFP) (green), for SgII (red) and for microtubule‐associated protein 2 (MAP2) (blue). (b) Zoomed area in the box shown in (a). (c) Pearson's coefficient quantification. (d) Mander's coefficient quantification. (e) Example images of CgA/B^−/−^ and control hippocampal neurons (DIV19) over‐expressing NPY‐pHluorin stained for GFP (green), MAP2 (blue), and SMI312 (red). (f) Zoomed areas in the boxes shown in (e). (g) Averaged histograms of the NPY‐pHluorin puncta intensities of neurons of both genotypes. Inset: Same histograms plotted separately. (h) Quantification of the mean NPY‐pHluorin puncta intensity per neuron. (i) Quantification of the number of NPY‐pHluorin puncta per neuron. (j) Mean number of NPY‐pHluorin per μm dendrite per neuron. (k) Mean number of NPY‐pHluorin puncta against the distance from the soma. (Data shown as mean ± SEM. Control *n* = 29 neurons and CgA/B^−/−^ *n* = 29 neurons from three independent cultures. (h and j) MW‐U test ***p = 0.0007. (i) Student's *t*‐test.

We also assessed DCV loading by expressing the pH‐sensitive DCV cargo reporter, NPY‐pHluorin, which targets DCVs in primary hippocampal neurons (van de Bospoort *et al*. 2012; Farina *et al*. 2015). As shown in Fig. 4(a–d), 81% of NPY‐pHluorin signal colocalized with SgII and 76% of SgII signal colocalized with NPY‐pHluorin (Pearson's coefficient was 0.8, Fig. 4c and d), confirming that NPY‐pHluorin targets to DCVs.

CgA/B^−/−^ and control hippocampal neurons expressing NPY‐pHluorin were stained for green fluorescence protein, MAP2, and SMI312 (Fig. 4e and f). NPY‐pHluorin puncta were analyzed using the same procedure described before for SgII (see above). The frequency distribution of the intensity of NPY‐pHluorin puncta was shifted to the left (less signal) in the CgA/B^−/−^ neurons (Fig. 4g), suggesting that CgA/B^−/−^ neurons contained puncta with, on average, lower signal than in controls. The mean intensity of NPY‐pHluorin puncta per neuron was reduced by 33% in CgA/B^−/−^ neurons (Fig. 4h). In addition, the puncta area was slightly smaller (7% reduction) in CgA/B^−/−^ (Control: 0.720 ± 0.014 μm^2^ and CgA/B^−/−^: 0.668 ± 0.018 μm^2^, MW‐U‐test *p* = 0.0189). However, the mean number of NPY‐pHluorin puncta (Fig. 4i), the mean number of puncta per micrometer of neurite (Fig. 4j) and the distribution of the puncta in the neurons (Fig. 4k) was the same in both genotypes. In summary, the number of NPY‐pHluorin puncta is the same in both genotypes; however, NPY‐pHluorin intensity per puncta is decreased by 33% in the CgA/B^−/−^ neurons.

### DCV cargo reporter NPY‐pHluorin reveals slightly less abundant fusion events in the absence of Cgs

**Figure 5:**
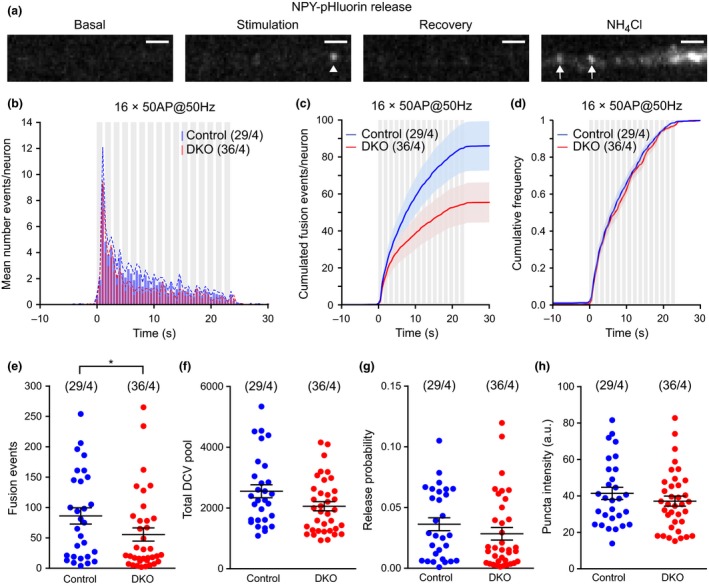
Dense‐core vesicle (DCV) fusion events are less frequent, but fusion probability is normal in the absence of Cgs. (a) Example of a neuropeptide Y (NPY)‐pHluorin release event (arrowhead) in a control neurite and the unquenching of all the DCVs present in the area upon NH_4_Cl puff (arrows). Scale bar: 2 μm. (b) Averaged frequency distribution of the events against the time of recording. SEM plotted as dotted line. The stimulation is shown in gray (16 bursts of 50 action potentials at 50 Hz). (c) Averaged cumulated fusion events per neuron. (d) Normalized cumulated fusion events. (e) Quantification of the total number of fusion events per neuron. (f) Total DCV pool per neuron calculated from NH_4_Cl puff signal. (g) Fusion probability of DCVs of both genotypes. (h) NPY‐pHluorin puncta intensity in the NH_4_Cl puff. (Data are shown as mean ± SEM. MW‐U test *p = 0.0477. Control *n* = 29 neurons and CgA/B^−/−^ *n* = 36 neurons from four independent cultures. (e, g, and h) MW‐U test. (f) Student's *t*‐test.

NPY‐pHluorin was used as a live DCV marker to analyze DCV fusion as described before (van de Bospoort *et al*. 2012; Farina *et al*. 2015). The low pH inside DCVs quenches the NPY‐pHluorin signal until fusion with the plasma membrane occurs and pHluorin is unquenched, producing a rapid fluorescence increase. DCV fusion was triggered using field stimulation (bursts of 50 action potentials, APs, at 50 Hz; for more details, see Materials & Methods, Fig. 5a). At the end of the protocol, an NH_4_Cl puff was applied to unmask the complete DCV pool (Fig. 5a). Fusion events were defined as a sharp increase in fluorescence signal of at least two times above the standard deviation of the background. DCV fusion was observed in 93% of CgA/B^−/−^ and 90% of the control neurons. Non‐secreting neurons were excluded from further analysis. In both genotypes, DCV fusion was strictly activity/Ca^2+^‐dependent. Approximately, 98% of the events occurred during the stimulation period (Fig. 5b) as observed before (van de Bospoort *et al*. 2012; Farina *et al*. 2015). Most of the events occurred in the first two bursts of the stimulation protocol followed by a reduction in the frequency of fusion events in subsequent bursts. The mean frequency histograms of the fusion events showed a similar distribution in both genotypes (Fig. 5b). The total number of events was reduced by 35% in the CgA/B^−/−^ neurons (Fig. 5c and e), while the fusion kinetics were similar in both genotypes (Fig. 5d). The total pool of DCVs was calculated using the images recorded during the NH_4_Cl puff (all pH‐sensitive fluorescence; for details, see Material and Methods). A tendency toward a reduced total DCV pool was observed in CgA/B^−/−^ neurons (approximately 20%, *n* = 29, 36 neurons, MW‐U *p* = 0.0686, n.s.) (Fig. 5f), which can be explained by the fact that CgA/B^−/−^ neurons were slightly smaller (11%, MW‐U *p* = 0.2272 n.s.) and contained slightly reduced number of DCVs per μm neurite (10%, MW‐U *p* = 0.4401 n.s.) (Figure S2b–d). In addition, a tendency toward a reduced DCV fusion probability was observed in CgA/B^−/−^ neurons (22%, MW‐U *p* = 0.1275 n.s., Fig. 5g). The intensity of the NPY‐pHluorin puncta, unmasked upon NH_4_Cl puff, tended to be reduced in CgA/B^−/−^ neurons (10%, MW‐U *p* = 0.3874) (Fig. 5h). Hence, DCV fusion was significantly reduced in CgA/B^−/−^ neurons, potentially because of small added differences in the size of the neurons, the number of DCVs per micrometer neurite, and their fusion probability.

### The duration and amplitude of DCV fusion events are normal in the absence of Cgs

**Figure 6:**
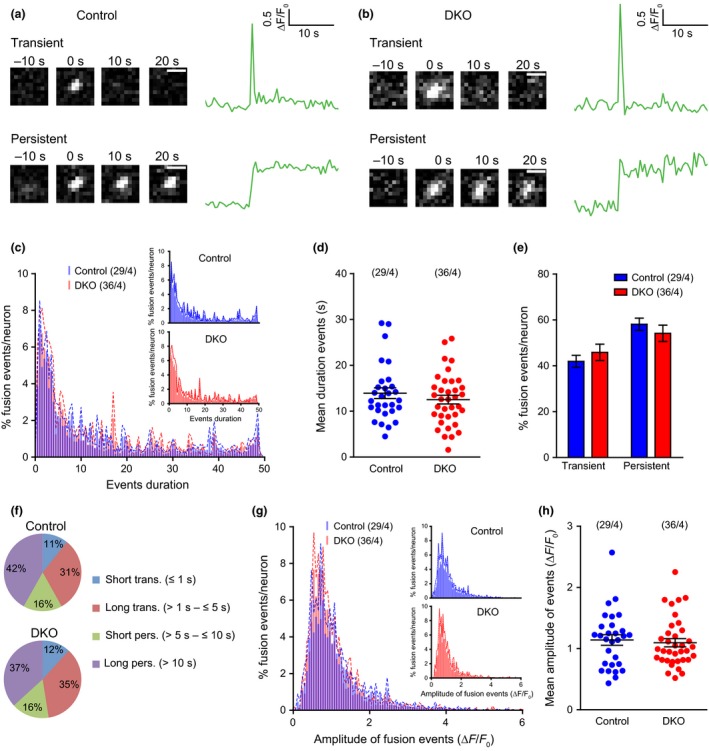
Duration and amplitude of fusion events is normal in the absence of Cgs. (a) Sequential frames showing examples of two types of neuropeptide Y (NPY)‐pHluorin release events in control neurons. Scale bar: 1 μm. Quantification of each event is shown as ΔF/F_0_. (b) Like in (a) but for CgA/B^−/−^ neurons. (c) Averaged histograms of the duration of the NPY‐pHluorin events. Inset: Same histograms plotted separately. (d) Mean duration of the events per cell. (e) Percentage of transient versus persistent events in both genotypes. (f) Classification of the events depending on the duration: short transient (≤1 s), long transient (> 1 s to ≤ 5 s), short persistent (> 5 s to ≥ 10 s), and long persistent (> 10 s). (g) Averaged histograms of the amplitude of the NPY‐pHluorin events. Inset: Same histograms plotted separately. (h) Quantification of the mean amplitude of the events per neuron. Data are shown as mean ± SEM. Control *n* = 29 neurons (3321 events) and CgA/B^−/−^ *n* = 36 neurons (2480 events) from four independent cultures. (d and h) MW‐U test. (e) Student's *t*‐test.

During the analysis of NPY‐pHluorin fusion events, different types of events (transient and persistent) were observed, as shown before (de Wit *et al*. 2009) (Fig. 5a and b). Transient events represent two types of events: complete fusion followed by diffusion of the cargo into extracellular space or incomplete fusion (‘kiss & run’) followed by re‐acidification of the vesicles. The persistent events represent stable deposits of cargo at the cell surface or (rarely) vesicles that reseal but fail to re‐acidify rapidly (de Wit *et al*. 2009). Since changes in the vesicle core matrix because of the absence of Cgs could affect fusion kinetics and/or the ratio of complete/incomplete fusion, we compared the duration of fusion events between CgA/B^−/−^ and control groups. The frequency distribution of the event duration was similar in both groups (Fig. 6c) and the mean event duration per cell was not different (Fig. 6d). Events were classified as transient and persistent (Fig. 6e) and subclassified depending on the duration of the events. Both genotypes showed similar percentages of the different events types (Fig. 6f). Taken together, these data suggest that the duration of single fusion events is not affected by the absence of Cgs.

To assess further whether the loading of vesicles is impaired by the absence of Cgs (in addition to the assays described above), we analyzed the amplitude of individual fusion events (ΔF/F0). The frequency distribution of the amplitudes of all events of both genotypes was similar (Fig. 6g). The mean amplitude of the events per neuron was not different between the two genotypes (Fig. 6h). In conclusion, the loading of DCVs with NPY‐pHluorin, as assessed by fusion amplitudes, is not affected by the absence of Cgs.

## Discussion

Neuronal DCVs are acidic organelles, 50–200 nm in size, which travel through the axons and dendrites in both anterograde and retrograde directions. DCVs contain typical cargo such as SgII and can store established DCV‐reporters such as NPY‐pHluorin/mCherry. Neuronal DCVs can release their cargo by exocytosis upon a robust stimulation protocol (16 bursts of 50 action potentials at 50 Hz), as it has been previously described in (de Wit *et al*. 2006, 2009; van de Bospoort *et al*. 2012; Farina *et al*. 2015).

Our aim was to analyze the role of Cgs in cargo sorting, DCV biogenesis, and exocytosis in hippocampal neurons. We found no changes in the main parameters to indicate a role for Cgs in these processes: the morphology and synapse density of cultured CgA/B^−/−^ neurons was normal; these neurons and their synapses contained a normal number of DCVs, with normal size and core; a RSP cargo reporter accumulated normally in these DCVs and their fusion characteristics showed no major defects. The only (minor) changes were observed in antibody staining for the cargo reporter, NPY‐pHluorin, which showed a 33% reduced intensity per punctum, and in the DCV fusion assay, which showed 35% less fusion events in CgA/B^−/−^ neurons. However, the first effect is contradicted by two other datasets in this study: the intensity of endogenous cargo SgII was not reduced and the pH‐sensitive NPY‐pHluorin signal per punctum (upon dequenching with ammonium in living neurons) was also unaltered; and the second effect may be explained by small (± 10%) differences in some of the underlying parameters (see Results and below). Hence, taken together, we conclude that these two minor differences are most likely explained by random experimental variation, given the fact that most central parameters are unaltered.

### DCV morphology depends on Cgs to a different extent in different tissues

No differences in DCV size or appearance were observed in CgA/B^−/−^ neurons (Fig. 3), but in chromaffin cells, granule diameter and core diameter were reduced (Figure S3b and c). While no other studies have analyzed DCV morphology in neurons before, conflicting conclusions were reached in studies on the morphology of chromaffin cell granules. Two studies on CgA‐single KO mice (Mahapatra *et al*. 2005; Pasqua *et al*. 2016) (the same mutant used in this study) showed a reduction in granule diameter, but no changes were found in a third study using a different CgA‐KO line (Hendy *et al*. 2006). Transgenic mice with decreased CgA levels (Kim *et al*. 2005) showed 20–38% increase in the chromaffin granules size. A previous study on CgA/B^−/−^ mice (the same mutant used in this study), described qualitative alterations in the secretory vesicles of the CgA/B^−/−^ mice such as enlargement of the vesicles and a more dispersed core (Díaz‐Vera *et al*. 2012). However, quantitative measurements were not performed in that study. Finally, insulin granules in β‐cells are the only secretory vesicles measured in CgB‐single KO mice (the same mutants as used in this study) and no differences were reported (Obermüller *et al*. 2010). These conflicting results can be partly explained by the different size of the ‘halo’, the DCV lumen surrounding the dense core. The granules in cryopreserved adrenals show a much smaller halo than chemically fixed adrenals (de Wit *et al*. 2009; Man *et al*. 2015), suggesting that the halo size is highly affected by different fixation and dehydration procedures used in electron microscopy. We report that the dense core itself is smaller in CgA/B^−/−^ adrenals, consistent with the reports on dense core size in CgA‐depleted chromaffin cells (Figure S3b, Kim *et al*. 2005; Pasqua *et al*. 2016). In contrast, the size of the dense core in CgA/B^−/−^ neurons remained unaffected, which is also observed in granules of β‐cells lacking CgB (Fig. 3; Obermüller *et al*. 2010). The data suggest that the participation of Cgs in dense core size is tissue specific. We have observed a tendency of increased expression of SgIII (non‐significant) in CgA/B^−/−^ brains that might assist in compensating for the absence of Cgs and preserving dense core size unaffected in the absence of Cgs in some cell types. Clearly, many differences exist in (genetic) models, sample preparation, fixation, quantitative analysis, and possibly other factors exist among these studies. In addition, none of the published studies included rescue experiments to test if acute Cg expression reverts the observed changes. Taken together, we conclude that although the absence of Cgs leads to a decrease in the number and size of chromaffin DCVs, this is not the case in hippocampal neurons.

### Cgs are not required for cargo sorting and DCV biogenesis in hippocampal neurons

The number of DCVs in CgA/B^−/−^ neurons per synapse was unaltered and the amount of RSP cargo reporter in acidic compartments (DCVs) was also normal. Previous studies have shown that expression of Cgs promotes sorting of cargo to the RSP, for instance increased pro‐opiomelanocortin in AtT20 cells (Natori and Huttner 1996), and is even sufficient for the formation of DCV‐like structures in non‐secretory cells (Kim *et al*. 2001; Huh *et al*. 2003; Stettler *et al*. 2009; Dominguez *et al*. 2014). Conversely, expression of Cg antisense RNAs in PC12 cells led to profound loss of DCVs (Kim *et al*. 2001; Huh *et al*. 2003) and fewer chromaffin granules in antisense‐CgA transgenic mice (Kim *et al*. 2005) and in CgA‐KO mice (~ 41%, same mutant used in this study) (Mahapatra *et al*. 2005; Pasqua *et al*. 2016). Together, these data led to the conclusion that Cgs are ‘on/off’ switches controlling DCV biogenesis (Kim *et al*. 2001; Huh *et al*. 2003). Hence, the strong prediction was that the number of DCVs would be reduced, also in CNS neurons. The fact that this was not observed in hippocampal neurons is difficult to explain, also because approaches to assess the function of Cgs have been different, but at least two possible explanations may be considered. First, unlike PC12 cells, chromaffin cells, and AtT20 cells, neurons may contain a more precise regulation of expression of proteins to avoid the ablation of the RSP. A recent study showed that some Cgs are selectively excluded from certain populations of neurons: CgB is absent in hippocampal interneurons (Ramírez‐Franco *et al*. 2016). Secretogranins II–IV are the most likely candidates, to provide redundant functionality. We have studied their expression by qRT‐PCR in the brains of CgA/B^−/−^ and WT mice, but we found no differences in their expression, except a tendency of over‐expression of SgIII (non‐significant) that could replace the role of CgA and CgB. Even without compensatory up‐regulation of expression of other members of the Cgs‐Sgs family, their presence could functionally compensate for the absence of Cgs in CgA/B^−/−^ hippocampal neurons. In a proteomic study performed in DKO chromaffin granules (Díaz‐Vera *et al*. 2012), significant amounts of fibrinogens were found only in the granules from DKO mice but not in WT mice, suggesting that other proteins unrelated with the Chromogranin‐Secretogranin family could also compensate the absence of Cgs in chromaffin cells. Second, antisense experiments are known to suffer from off‐target effects and most published studies to date have not controlled for that using expression of antisense‐resistant versions of Cgs. Together these data suggest that Cgs are dispensable for cargo sorting and DCV biogenesis.

### Cgs are not required for DCV fusion in hippocampal neurons

The total number of fusion events was reduced by 35% in the CgA/B^−/−^ neurons (Fig. 5c and e). However, the fusion kinetics (Fig. 5d) and probability (Fig. 5g) were not changed. This effect is significant and may be a bona fide phenotype of Cgs loss, but may also be an added effect of small experimental variations in the underlying parameters: CgA/B^−/−^ neurons tended to be slightly smaller (not significant, Figure S2d), contained slightly fewer DCVs per μm neurite (not significant, Figure S2e) and tended to have slightly lower fusion probability (not significant, Fig. 5g). Furthermore, it is generally accepted that DCV fusion events are initiated by molecules outside the vesicle, together with molecules at the target membrane, and there is no conceptual link to molecules inside the vesicle, until the fusion pore has opened. After fusion pore opening, vesicle constituents like Cgs may affect release properties and the kinetics of catecholamine release were indeed affected in the absence of Cgs (Montesinos *et al*. 2008; Díaz‐Vera *et al*. 2010, 2012) and over‐expression of CgA in PC12 cells increased the speed of the initial release phase (Dominguez *et al*. 2014). However, in this study in hippocampal neurons using live‐cell imaging epifluorescence in NPY‐pHluorin loaded DCVs, parameters relating to fusion kinetics (event duration, amplitude) were unaltered in the absence of Cgs (Fig. 6). Taken together we conclude that the 35% reduction in DCV fusion events in CgA/B^−/−^ neurons is most likely the consequence of random experimental variation in the underlying parameters. In any case, most parameters related to DCV fusion were normal and fusion events were still abundant in the absence of Cgs. Therefore, we conclude that Cgs are not essential for DCV fusion in CgA/B^−/−^ hippocampal neurons.

## Supporting information


**Figure S1.** Calcium imaging protocol.
**Figure S2.** Total DCV pool calculation.
**Figure S3.** Chromaffin dense‐core diameter, granule diameter, and number of DCVs is decreased in CgA/B^−/−^ adrenal glands.
