# Supplementary material for: Dense‐core vesicle biogenesis and exocytosis in neurons lacking chromogranins A and B
Source: J Neurochem. 2017 Dec 27;144(3):241–54. doi: 10.1111/jnc.14263 (PMC5814729; doi:10.1111/jnc.14263)
Supplement: Supplementary file 1 — Figure S1. Calcium imaging protocol. Figure S2. Total DCV pool calculation. Figure S3. Chromaffin dense‐core diameter, granule diameter, and number of DCVs is decreased in CgA/B−/− adrenal glands. [file JNC-144-241-s001.pdf]

**Supplementary figures**

# Dense-Core Vesicle biogenesis and exocytosis in neurons lacking Chromogranin A and B.

Natalia Dominguez[1], Jan RT van Weering[1], Ricardo Borges[3], Ruud F Toonen[2] and Matthijs Verhage[1, 2].

[1]Department of Clinical Genetics and [2]Functional Genomics, Center for Neurogenomics and Cognitive Research (CNCR), VU University Amsterdam and VU University Medical Center (VUmc), Amsterdam, Netherlands, [3]Unidad de Farmacología, Facultad de Medicina, Universidad de la Laguna, Tenerife, Spain,

Figure S1

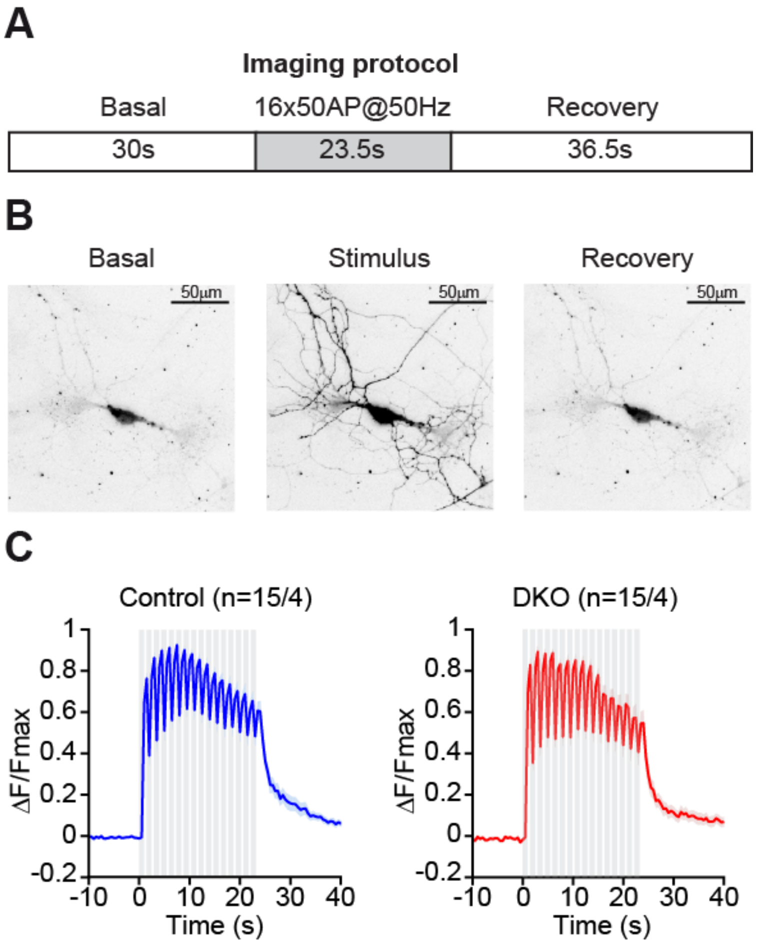


Dominguez et al.

Figure S2

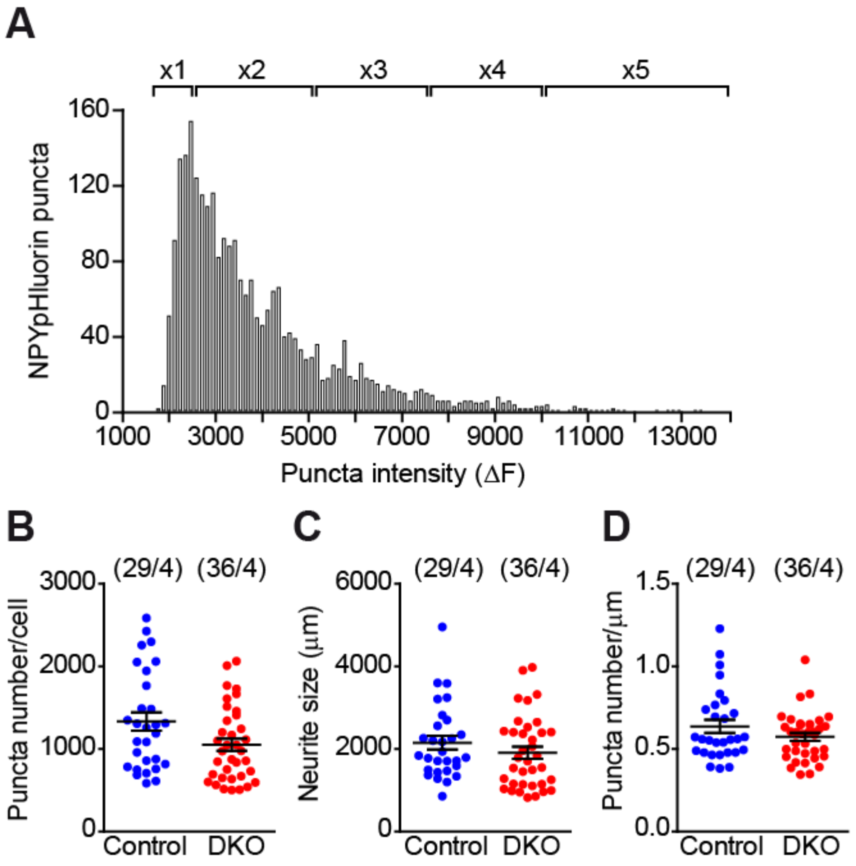


Dominguez et al.

Figure S3

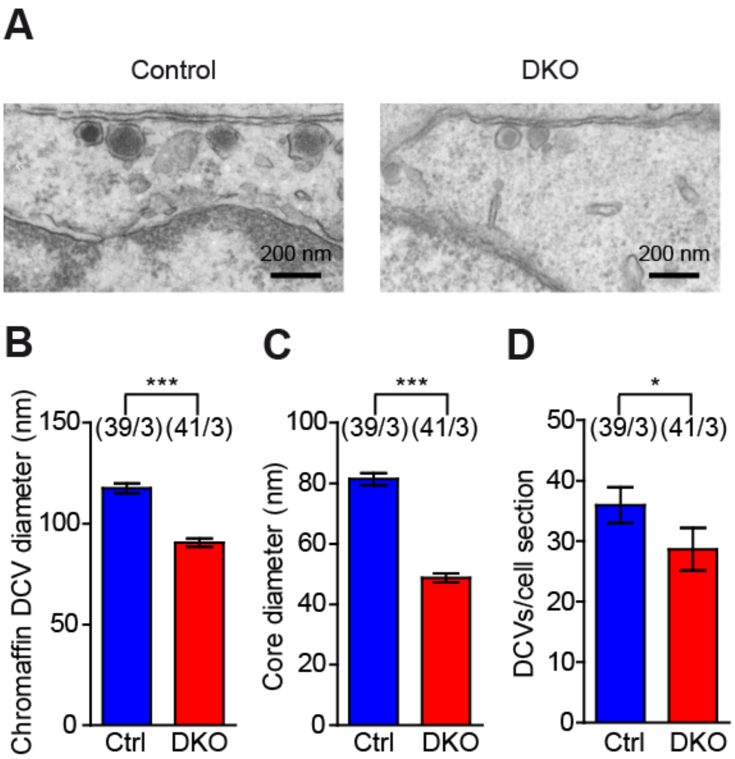


Dominguez et al.

**Figure S1: Calcium imaging protocol**

A) Calcium imaging protocol. B) Example frames of the time-lapses during the different stages of the calcium imaging protocol. C) Quantification of the calcium influx and efflux of CgA/B$^{-/-}$ and control neurons. (Data shown as mean ± SEM. Control n = 15 neurons and CgA/B$^{-/-}$ n = 15 neurons from 4 independent cultures)

**Figure S2: Total DCV pool calculation**

A) Histogram of NPY-pHluorin intensity puncta from a cell after $NH_4Cl$ puff with an estimation of vesicles per puncta. B) Quantification of the number of NPY-pHluorin puncta per cell after the $NH_4Cl$ puff. C) Quantification of neurite size. D) Quantification of number of NPY-pHluorin puncta per μm of neurite.

**Figure S3: Chromaffin dense-core diameter, granule diameter and number of DCVs is decreased in CgA/B$^{-/-}$ adrenal glands.**

A) Example electron micrographs of adrenal glands from P1 CgA/B$^{-/-}$ and control mice. B) Quantification of the mean diameter of chromaffin DCVs. C) Quantification of the mean diameter of dense-cores. D) Quantification of the number of DCVs per cell section. (Data shown as mean ± SEM. MW-U test ***p < 0.0001, *p = 0.0256. Control n = 40 chromaffin cells and CgA/B$^{-/-}$ n = 40 chromaffin cells from 3 pups per genotype from 3 independent nests).
